# Supplementary material for: Asynchronous magnetic resonance elastography: Shear wave speed reconstruction using noise correlation of incoherent waves
Source: Magn Reson Med. 2022 Oct 27;89(3):990–1001. doi: 10.1002/mrm.29502 (PMC9792433; doi:10.1002/mrm.29502)
Supplement: Supplementary file 1 — DATA S1 MATLAB code used to generate the numerical simulations of Figure 1 [file MRM-89-990-s002.zip › k-Wave/helpfiles/example_ivp_axisymmetric_simulation.html]

Simulations In An Axisymmetric Coordinate System Example (k-Wave)


# Simulations In An Axisymmetric Coordinate System Example

This example provides a simple demonstration of using k-Wave for the simulation and detection of the pressure field generated by an initial pressure distribution within an axisymmetric coordinate system. It builds on the Homogeneous Propagation Medium and Heterogeneous Propagation Medium examples.

- Open the file in the MATLAB Editor
- Run the file in MATLAB

## Contents

- Creating the k-space grid and defining the medium properties
- Defining the initial pressure distribution and sensor mask
- Running the simulation

## Creating the k-space grid and defining the medium properties

Axisymmetric simulations are performed in an analogous fashion to those in two-dimensions. However, in an axisymmetric coordinate system, the x-dimension corresponds to the axial direction, while the y-dimension corresponds to the radial direction, as shown below. The coordinate system is rotationally symmetric about the x-axis, thus a point on the y-axis corresponds to a continuous circle in 3D space.

Once the grid parameters have been defined, the medium discretisation is performed by `kWaveGrid` in an identical manner to 2D simulations. However, in the radial dimension, the first grid point now corresponds to the grid origin, i.e., `y = 0`. In comparison, for `kspaceFirstOrder2D`, the Cartesian point `y = 0` is in the middle of the computational grid. For a heterogeneous acoustic propagation medium, the medium properties are given as two-dimensional matrices the same size as the computational grid. In this example, the properties are divided into two half-spaces.

```
% create the computational grid
Nx = 128;           % number of grid points in the axial (x) direction
Ny = 64;            % number of grid points in the radial (y) direction
dx = 0.1e-3;        % grid point spacing in the axial (x) direction [m]
dy = 0.1e-3;        % grid point spacing in the radial (y) direction [m]
kgrid = kWaveGrid(Nx, dx, Ny, dy);

% define the properties of the propagation medium
medium.sound_speed = 1500 * ones(Nx, Ny);       % [m/s]
medium.sound_speed(Nx/2:end, :) = 1800;         % [m/s]
medium.density = 1000 * ones(Nx, Ny);           % [kg/m^3]
medium.density(Nx/2:end, :) = 1200;             % [kg/m^3]
```

## Defining the initial pressure distribution and sensor mask

As in two-dimensions, the initial pressure distribution is set using a matrix which contains the initial pressure values for each grid point within the computational domain. However, for the axisymmetric code, the initial pressure is rotationally symmetric about the first column in the matrix. For example, if the initial pressure is set to a single grid point in the first column (i.e., on the x-axis for y = 0), this corresponds to setting a point source within a 3D simulation. Conversely, if the initial pressure is set to a single grid point in any other column, this corresponds to setting a ring in 3D. In this example, the initial pressure is set in the shape of half a disc, which corresponds to a ball in 3D.

```
% create initial pressure distribution in the shape of a disc - this is
% generated on a 2D grid that is doubled in size in the radial (y)
% direction, and then trimmed so that only half the disc is retained
source.p0 = 10 * makeDisc(Nx, 2 * Ny, Nx/4 + 8, Ny + 1, 5);
source.p0 = source.p0(:, Ny + 1:end);
```

Again the sensor mask, which defines the locations where the pressure field is recorded at each time-step, can be given as a list of Cartesian coordinates, a binary mask, or the grid coordinates of two opposing corners of a rectangle. In this example, a Cartesian sensor mask in the shape of a circle is defined. A visualisation of the initial pressure and the sensor mask is shown below. Note, for the axisymmetric code, in the radial (y) direction, the perfectly matched layer (PML) is only applied to the outer edge of the domain. The default PML size is 20 grid points.

```
% define a Cartesian sensor mask with points in the shape of a circle
sensor.mask = makeCartCircle(40 * dx, 50);

% remove points from sensor mask where y < 0
sensor.mask(:, sensor.mask(2, :) < 0) = [];
```


## Running the simulation

The computation is started by passing the four input structures, `kgrid`, `medium`, `source`, and `sensor` to `kspaceFirstOrderAS`. By default, a visualisation of the propagating wave-field and a status bar are displayed. As the function runs, status updates and computational parameters are printed to the command line.

```
Running k-Wave simulation...
  start time: 26-Apr-2018 10:42:39
  reference sound speed: 1800m/s
  dt: 16.6667ns, t_end: 9.5333us, time steps: 573
  input grid size: 128 by 64 grid points (12.8 by 6.4mm)
  maximum supported frequency: 7.5MHz
  smoothing p0 distribution...
  calculating Delaunay triangulation...
  precomputation completed in 0.30384s
  starting time loop...
  estimated simulation time 3.14s...
  simulation completed in 3.6796s
  total computation time 4.043s
```

When the time loop has completed, the function returns the recorded time series at each of sensor points defined by `sensor_mask`. The ordering is again dependent on whether a Cartesian or binary sensor mask is used. A visualisation of the recorded sensor data is given below.
